# Establishing a distributed system for the simple representation and integration of diverse scientific assertions

**DOI:** 10.1186/2041-1480-1-S1-S5

**Published:** 2010-06-22

**Authors:** Matthias Samwald, Holger Stenzhorn

**Affiliations:** 1 Digital Enterprise Research Institute (DERI), Galway, Ireland

## Abstract

**Background:**

Information technology has the potential to increase the pace of scientific progress by helping researchers in formulating, publishing and finding information. There are numerous projects that employ ontologies and Semantic Web technologies towards this goal. However, the number of applications that have found widespread use among biomedical researchers is still surprisingly small. In this paper we present the aTag (‘associative tags’) convention, which aims to drastically lower the entry barriers to the biomedical Semantic Web. aTags are short snippets of HTML+RDFa with embedded RDF/OWL based on the Semantically Interlinked Online Communities (SIOC) vocabulary and domain ontologies and taxonomies, such as the Open Biomedical Ontologies and DBpedia. The structure of aTags is very simple: a short piece of human-readable text that is ‘tagged’ with relevant ontological entities. This paper describes our efforts for seeding the creation of a viable ecosystem of datasets, tools and services around aTags.

**Results:**

Numerous biomedical datasets in aTag format and systems for the creation of aTags have been set-up and are described in this paper. Prototypes of some of these systems are accessible at http://hcls.deri.org/atag

**Conclusions:**

The aTags convention enables the rapid development of diverse, integrated datasets and semantically interoperable applications. More work needs to be done to study the practicability of this approach in different use-case scenarios, and to encourage uptake of the convention by other groups.

## Background

A common challenge faced by biomedical researchers and clinicians is to quickly get an overview of publications and database entries for a certain biomedical topic, and to identify relevant, valid facts, research trends and contradictory findings from diverse sets of information sources. From here on, we will refer to this process as “key assertion integration”.

Novel information technologies might enable researchers to conduct these tasks in a more efficient and reliable manner. For example, Semantic Web technologies and ontologies hold the promise to enable the creation of smarter software systems that facilitate key assertion integration through better structuring of information, shared standards, clear semantics and global interlinking of data. These technologies have undergone remarkable progress in recent years. For instance, the Open Biomedical Ontologies (OBO) Foundry [1] and the BioPortal offered by the National Center for Biomedical Ontology (NCBO) [2] make a vast array of biomedical ontologies of great detail and high quality available to the public. Large, integrated biomedical knowledge bases have been created using the Resource Description Framework (RDF) and the Web Ontology Language (OWL), such as the Neurocommons Knowledge Base [3,4] or the knowledge base [5] of the World Wide Web Consortium (W3C) Health Care and Life Science Interest Group (HCLSIG) [6]. The Linked Data community [7] created a global network of RDF/OWL resources that consists of billions of statements, with each resource being available for fine-grained access based on established web standards.

However, there is still a widely recognized lack of applications that empower end-users to access, add and link to this structured, ontology-based information on the web. While many current biomedical RDF/OWL resources offer a wealth of valuable information, their structures are often highly complex and heterogeneous. Even though diverse datasets are interlinked and similarly formalized through RDF/OWL, the complexity and stylistic differences between them make it hard for developers to create applications that are both user-friendly and flexible enough to work with them without extensive customization [8].

In this paper we introduce a convention for using existing Semantic Web standards, vocabularies and ontologies in a way that is decidedly less complex and easier to implement in diverse software applications. We call this convention *aTag* (‘associative tag’).

The hypothesis that drives the aTag developments is that semi-structured data can be sufficient to tackle many realistic biomedical use-cases, and that simpler data structures are easier to integrate, understand and use than more complex ones. This hypothesis is inspired by experiences we made with creating, integrating and using large-scale ontology-based information repositories in recent years. In the case of aTags, a set of ontological entities is used to describe biomedical statements without capturing the actual relationships (RDF properties) between those entities.

In this paper we describe our efforts to implement this convention in a variety of web applications and try to demonstrate that this simpler approach can nonetheless be used to tackle realistic problems associated with the task of key assertion integration. A prototype of the software described here is accessible at http://hcls.deri.org/atag

The aTag project is carried out in cooperation with the BioRDF task force [9] of the aforementioned HCLSIG and in cooperation with the “Hypotheses, Evidence & Relationships” (HypER) group [10,11].

## Results and discussion

### The anatomy of an aTag

**Figure 1:**
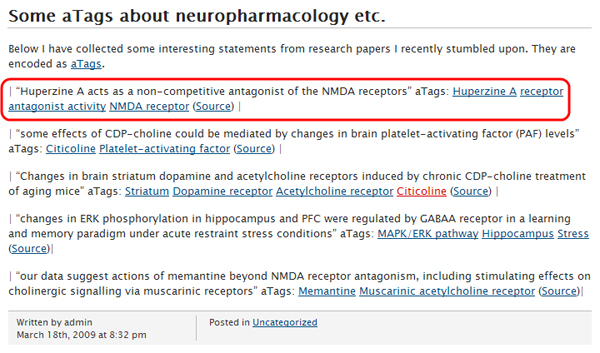
aTags embedded on the HTML page of a personal blog, viewed in a normal web browser. An exemplary aTag is highlighted in this figure, its source code and embedded RDF/OWL statements are detailed in this paper.

aTags are normally serialized as short snippets of HTML inside a web page. They contain embedded RDF/OWL statements, based on the new RDFa standard [12]. RDFa makes it possible to embed simple RDF/OWL statements or even entire ontologies within HTML documents, re-using existing HTML elements where possible. Thus human-readable and machine-readable information can be unified in a single document, making it easy to re-use and extend existing systems, such as web-based database front ends and content management systems. Figure 1 exemplifies how aTags embedded on a web page look like.

aTags do not depend on any new vocabularies but are based on reusing the popular SIOC vocabulary [13] combined with entities from established ontologies and taxonomies, such as OBO ontologies or DBpedia [14,15]. These entities are used to annotate short snippets of text containing assertions.

**Figure 2:**
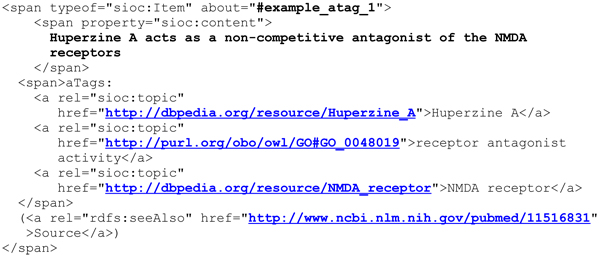
Representation of an exemplary aTag in HTML syntax. The exact formatting and style of the aTag can be varied.

The HTML code of the aTag highlighted in Figure 1 is shown in Figure 2 (slightly simplified to improve readability).

**Figure 3:**
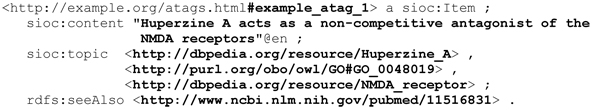
Representation of an exemplary aTag in Turtle syntax.

**Figure 4:**
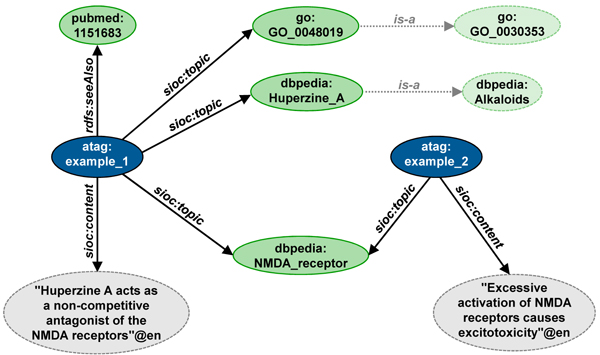
Representation of an exemplary aTag in Turtle syntax. A neighbouring aTag and parts of the entity hierarchy of DBpedia and the Gene Ontology are displayed as well, demonstrating the interconnectedness of aTags through shared identifiers.

Besides RDFa, aTags can also be encoded with other standard RDF serialisations such as RDF/XML or Turtle [16], although RDFa is recommended to facilitate accessibility through common web browsers. The RDF statements contained in the snippet of HTML from Figure 2 are shown in Figure 3 and Figure 4.

The number of ontological entities that can be used to ‘tag’ a specific aTag is not limited in any way. While the three tags used for the exemplary aTag shown above resemble a typical subject-predicate-object triple, the number of tags can also be smaller or larger, and the tags do not need to follow a subject-predicate-object pattern. This makes it possible to represent even complex statements without creating convoluted RDF graphs. For example, a statement such as “Protein A interacts with protein B in tissue C at time D” could be represented as an aTag that is tagged with five entities: ‘Protein A’, ‘molecular interaction’, ‘protein B’, ‘tissue C’, ‘time D’. Albeit not all relationships between the entities are explicitly encoded, a wide variety of queries could be answered with a dataset that is made up of aTags following this pattern. In comparison, trying to fully represent the statement above with explicit RDF triples (e.g., using the Basic Formal Ontology (BFO) [17] and the Relation Ontology [18]) would result in an RDF graph that would be quite complex and significantly more difficult to query.

Obviously, this type of representation cannot capture all the details of complex assertions. For example, a small interaction network containing more than two proteins that interact in more than one manner cannot be represented as an aTag without information loss. Such limitations to expressivity are consciously accepted, based on the rationale that in many scenarios the gain in utility by keeping things simple outweighs the loss in utility by limited expressivity.

### Tools for creating aTags

**Figure 5:**
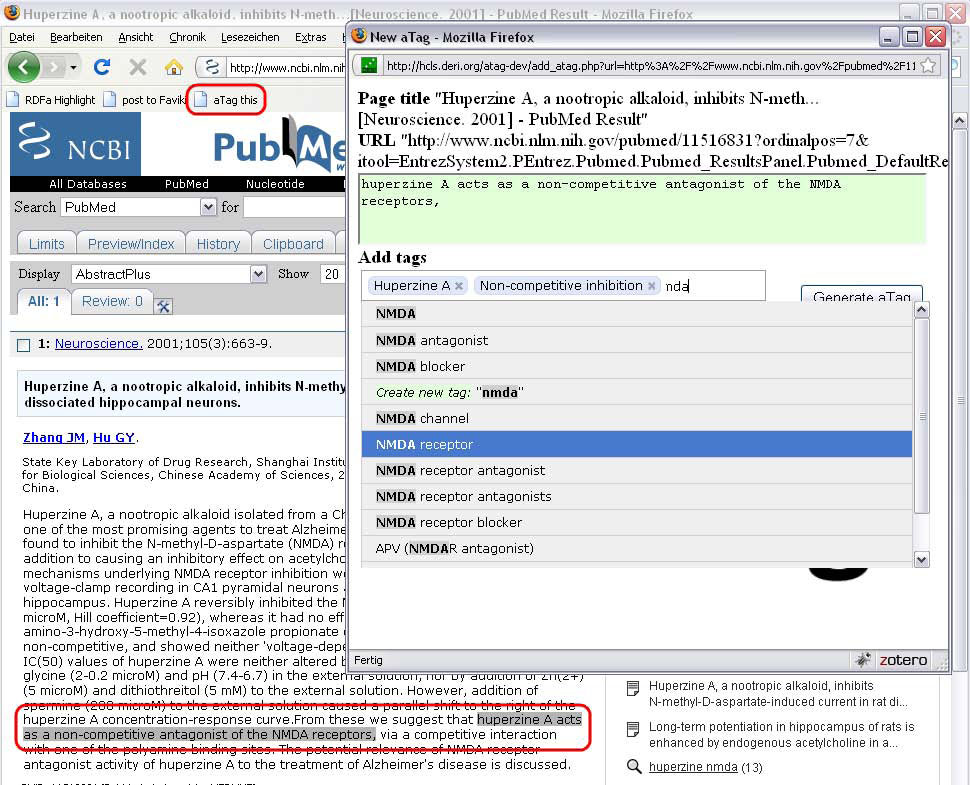
Creating an aTag out of a PubMed abstract inside the web browser, using the aTag bookmarklet. The simple statement “Huperzine A acts as a non-competitive antagonist of the NMDA receptor” is captured by tagging with the DBpedia entity ‘Huperzine A’, the OBO Gene Ontology entity ‘receptor antagonist activity’ and the DBpedia entity ‘NMDA receptor’.

Anybody with access to the web can quickly create aTags by adding the aTag bookmarklet to his or her web browser. (The bookmarklet is a bookmark with embedded Javascript that calls the aTag generator.) A user can navigate to any web page – s such as an abstract of an article on PubMed –, highlight a relevant statement in the text, and click on the bookmarklet. The aTag generator then captures the highlighted statement and allows the user to add and refine semantic ‘tags’ (entities from DBpedia and OBO), as exemplified in Figure 5. These tags capture the key entities mentioned in the statement in a machine-readable format, interlinking it with existing ontologies and Linked Data [7] resources. aTags generated by the bookmarklet are collected in the central ‘'aTag Pastebin’, but can also be copied over to other locations such as personal websites or blogs. It is also easily possible to configure the aTag bookmarklet to feed its output directly fed into third-party systems, such as content management systems and databases.

The RDFa embedded within the HTML is also carried along when parts of web documents are copied and pasted to other locations, making it possible to re-arrange and merge datasets with the ease of re-arranging free text in a What-You-See-is-What-You-Get (WYSWYG) editor.

### Data available in aTag format

To date, several biomedical datasets based on aTags are available (see Table 1). The data sources are also collected on a web page that can be found at [19]. All data sources listed here have also been integrated into the Health Care and Life Science Knowledge Base [5] and can be queried through a SPARQL endpoint.

**Table 1:** Currently available data sources in aTag format.

Data source	Description	Source of entities used for tagging
SIDER	Drug side-effect data. Size: 100,000 RDF statements.	DBpedia, ChEBI
PDSP Ki Database	Receptor-ligand interactions quantified by Ki value, emphasis on data about psychoactive substances.	SenseLab, ChEBI
PubMed Conclusion sections	Conclusion sections extracted from PubMed abstracts, annotated with Medical Subject Headings. Size: 1,8 million RDF statements.	SKOS version of Medical Subject Headings (MeSH)
Science Commons Text Annotation Service	Webservice that annotates sentences in PubMed abstracts with aTags	Gene Ontology, ChEBI, some smaller OBO ontologies, Uniprot and NCBI Taxonomy
aTag Pastebin	User-generated content, mostly curated from scientific texts on the web, created with the aTag bookmarklet. Contains curated statements in diverse domains such as pharmacology and neuroscience.	DBpedia

ChEBI: Chemical Entities of Biological Interest. MeSH: Medical Subjct Headings. PDSP: Psychoactive Drug Screening Programme. SKOS: Simple Knowledge Organisation System.

Several hundreds of thousands of aTags have been extracted from conclusion sections of PubMed abstracts. The SIDER dataset contains aTags that were generated out of a structured database, SIDER [20], describing associations between drugs and side-effects that were reported for these drugs. aTags have also been derived from the Psychoactive Drugs Screening Programme (PDSP) Ki Database, which contains quantitative data about ligand-receptor interactions [21], primarily of psychoactive substances.

**Figure 6:**
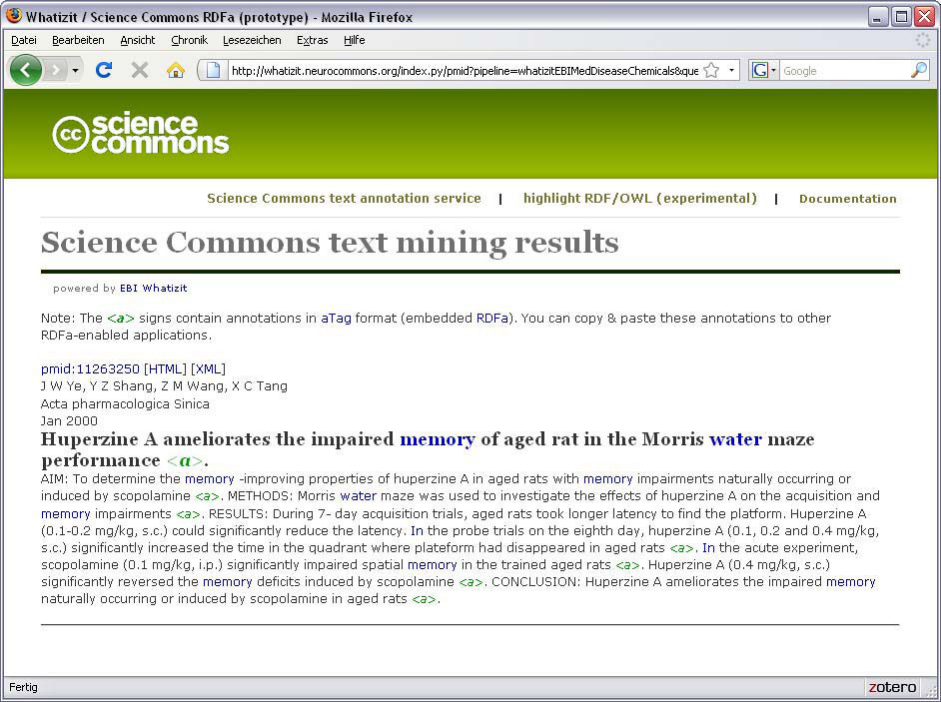
The Science Commons Text Annotation service can automatically generate aTags out of sentences in PubMed abstracts.

aTags can be automatically generated out of unstructured text on-the-fly by using the Science Commons Text annotation service [22]. In its current form, this service accepts a PubMed query or a PubMed ID, and returns the resulting PubMed abstracts as HTML where each sentence is annotated with an aTag (Figure 6). Entities in each sentence are recognized by calling the external Whatizit web service [23,24] offered by the European Bioinformatics Institute, linking the resulting aTags to the Gene Ontology, ChEBI, some smaller OBO ontologies, Uniprot and the NCBI Taxonomy. Functionality to submit free text to the web service is in preparation.

### Finding and Querying aTags

**Figure 7:**
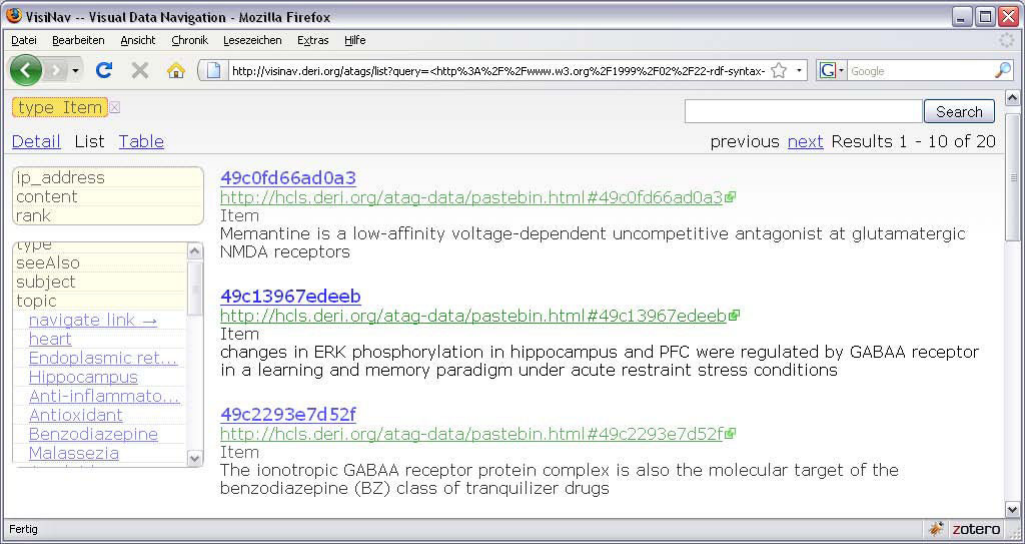
Faceted browsing of aggregated data from aTags, DBpedia and OBO ontologies on the web, crawled and visualized by Visinav.

Documents containing aTags can be found with Semantic Web search engines such as Sindice [25,26]. Collections of aTags that are located on a single web page can be easily made accessible for generic faceted browsing with SIMILE Exhibit [27]. Since aTags distributed over the web use shared identifiers, aTags at different locations are interlinked with each other, as well as with expressive domain ontologies and Linked Data resources. This makes it possible to search, explore and query aTags in a very sophisticated manner. This is exemplified by Visinav [28] (Figure 7), which crawls interlinked RDF/OWL on the web and allows for faceted searching over the aggregated data from these distributed resources.

**Figure 8:**
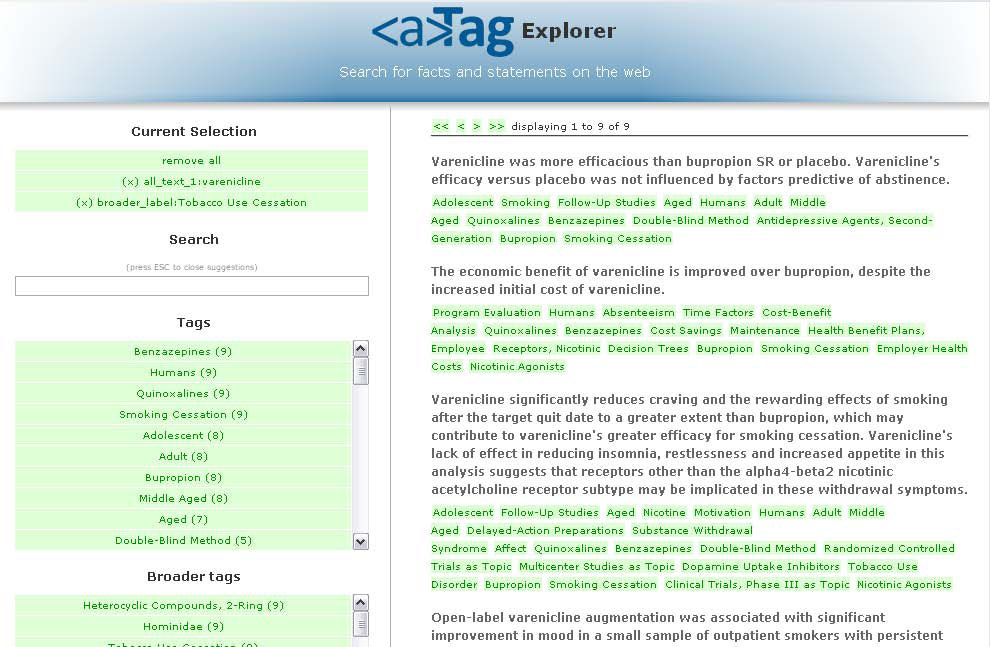
Navigating through key assertions from various data sources with the aTag Explorer web interface. Here, a user did a text search for the drug ‘varenicline’, then restricted results to those statements that deal with ‘Tobacco Use Cessation’ by selecting a facet value. The tags / facet values for each statement are terms from terminologies / ontologies such as MeSH and DBpedia. The ‘Broader tags’ for each statement are inferred by the system from these terminologies / ontologies. This makes it possible to identify links between statements that are not explicitly contained in the source literature and datasets.

A dedicated, user-friendly interface for convenient faceted browsing of aTags found on the web is the *aTag Explorer* (Figure 8). It is accessible through a web interface at [29] (currently supporting all browsers except Internet Explorer). The aTag Explorer allows users to navigate aTags through text search, narrowing and expanding result sets based on taxonomies/ontologies, and moving between entities that are connected by key assertions.

Preliminary results of using the aTag Explorer to answer realistic biomedical problems are encouraging, giving results of satisfying accuracy and coverage, even though the underlying datasets and text corpora are still very limited. The combination of aTags enriched with the background information drawn from taxonomy and ontology hierarchies makes it possible to search for scientific statements combining keyword search and exploratory search in an intuitive manner. To illustrate a typical search process with the aTag explorer, we can imagine a pharmaceutical researcher that wants to get a quick overview of products from the company Pfizer, including their types, mechanisms of action and side effects, and draw comparisons to drugs from competing pharmaceutical companies:

• The user types “Pfizer” into the search text field an hits enter (normal keyword search).

• The search results are more diverse than expected, including persons and institutions. The user selects ‘drug’ in the ‘broader tags’ facet to narrow down the result set (exploratory search).

• The result set is updated and shows several drugs developed or manufactured by Pfizer. The ‘tags’ facet now contains a list of names of these drugs, while the ‘broader tags’ facet contains a list of drug classes drawn from the underlying taxonomies / ontologies (e.g., out of the drugs in the current result set, two are antibiotics, one is a statin, et cetera). In scenarios like this one, these lists of facet values do not only serve the purpose of narrowing down search results, but can provide valuable information in themselves.

• Now, the users wants to retrieve more detailed information about a particular drug in the result set, Celecoxib, by clicking on the tag. All information in the knowledge base about Celecoxib is shown, including several reported side-effects for this medication. Celecoxib is classified as a ‘pyrazole’ in the ‘broader tags’ facet.

By applying the procedures of keyword search, inspecting ‘tags’ and ‘broader tags’, narrowing down on tags, shifting the focus by selecting related entities, and un-focusing the search by removing selected facet values, the researcher can now obtain information pyrazole drugs manufactured by other companies, and compare their side effects and indications.

Besides these general-purpose aTag search interfaces, there are also more specialized solutions under preparation. For example, Jun Zhao from the University of Oxford created a search interface for aTags specialized to herbal medicines [30].

### Related work

The SWAN Scientific Discourse Ontology [31,32] is currently applied in several biomedical projects. Its representation of scientific assertions resembles the aTag convention, but it also contains additional vocabulary, e.g., for the representation of agreement and disagreement between statements. SWAN has recently been mapped to SIOC as part of the work of the W3C Health Care and Life Science Interest group. We plan to make the developments around aTags compatible with SWAN and try to further interlink both efforts.

The aTags extracted from PubMed conclusion sections can be compared to a variety of sentence-based, whole-abstract search systems include I-HOP [33], Wikigenes [34] and MedEvi [35]. While these systems provide far better coverage, the search results contain a lot of unwanted noise produced by statements derived from introduction, methods and results sections of abstracts, producing results that are often not very relevant, unintelligible outside of the context of the entire text, or very redundant (e.g., introduction sections of abstracts often re-iterate the same fact again and again). In comparison, the statements derived by extracting conclusion sections seem to contain far less noise and might provide much better user satisfaction, even though coverage is drastically lower. Further research is needed to characterize these differences in a more objective and quantifiable manner.

## Conclusions

First experiences with creating interlinked datasets and interoperable applications based on the aTag convention were very positive. We are currently pursuing several threads of development, among them are:

• Converting additional data from existing structured, biomedical databases into aTags.

• Adding a ‘tag recommender’ function to the aTag generator by integrating the Open Biomedical Annotator web service [36] of NCBO.

• Creating a mapping between OBO ontologies and DBpedia.

A long-term goal of this work is to drive the integration of aTags or similar RDFa-based means for key assertion integration into the scientific publication process. First advances towards the establishment of ‘structured digital abstracts’ as part of biomedical publications have already been undertaken, as exemplified by the FEBS Letters experiment [37]. In this experiment, authors submitting papers to the molecular biology journal FEBS Letters were called on to submit a structured digital abstract alongside their manuscript, detailing molecular interactions reported in the paper in a machine-readable way. While this and other existing initiatives are important steps forward, they are still lacking interfaces that are easy to use for authors (leading to low compliance by users) and fail to use interlinked, standards-based and semantically rich formats (not realizing the full potential of the structured information). The use of simple, RDFa-based representation formats as outlined in this paper could help to drive the establishment of structured digital abstracts.

Readers that are interested in creating or consuming aTags to solve problems in their particular domains are encouraged to contact the authors of this paper.

## Methods

Technical details about the tools and datasets are described below.

The aTags generator is based on an Ajax interface and server-side PHP code. Apache Solr [38] (which is based on Apache Lucene [39]) is used for the autocompletion of entity tags. Solr/Lucene allows for optimizing the relevance of suggested tags by elaborate ranking based on contextual cues and ontological structures.

### Generation of aTags from existing structured databases

aTags were created from existing structured databases through PHP scripts that processed dumps of the original databases.

### Science Commons text annotation service

The Science Commons text annotation service is a RESTful web service written in Python. When invoked, it submits input to the Whatizit SOAP web service hosted at the European Bioinformatics institute. The XML result retrieved from the Whatizit service is parsed, ontology identifiers are mapped to URIs of the RDF/OWL versions of the respective ontologies, and a HTML+RDFa page is generated.

### Generation of aTags from PubMed conclusion sections

In a sizeable fraction of PubMed abstracts, the narrative of the abstract is clearly delineated by explicit section headers (*‘INTRODUCTION:’, ‘METHODS:’, ‘RESULTS:’, ‘CONCLUSIONS:’*). A search in PubMed reveals that ~ 1,7 million abstracts contain the words ‘conclusion’ or ‘conclusions’ (out of a total of ~ 19 million citations indexed in PubMed). Most of these abstracts really do contain a clearly delineated conclusion section. This means that a huge corpus of biomedical abstracts with explicit conclusion sections exists, covering a broad area of knowledge domains.

aTags were generated from PubMed conclusion section by creating a PHP script that accomplished the following tasks:

1) Retrieve PubMed abstracts containing conclusion sections for a certain query. The script could process all ~1,7 million abstracts with explicit conclusion sections, but for this trial, a more restrictive query was chosen that retrieves abstracts about emotion and cognition:

("conclusion"[Title/Abstract] OR "conclusions"[Title/Abstract]) AND (antidepressant OR "Emotions"[Mesh] OR "Behavioral Symptoms"[Mesh] OR "Mood Disorders"[Mesh])

This yields 58.000 results. Note that removing the constraint for ‘conclusion’ or ‘conclusions’ in this query would increase the number of results to 430.000, which means that roughly 1/7th of the abstracts for this topic contain an explicit conclusion sections.

2) Abbreviations that are locally defined in each abstract are expanded to their long forms using the Schwartz & Hearst algorithm [40]. In most abstracts, abbreviations are introduced in the introduction section, e.g.:

„ INTRODUCTION: Seasonal affective disorder (SAD) is common in ...“

while the conclusion sections contain lots of these abbreviated forms that tend to be unintelligible when only the conclusion sections are viewed in isolation, e.g.:

„ CONCLUSIONS: This study shows that SAD is effectively treated with ...“

The script recognizes local abbreviations and expands them, making the conclusion sections better intelligible. E.g., after processing the conclusion now reads

“CONCLUSIONS: This study shows that Seasonal affective disorder is effectively treated with …“

3) The conclusion sections are then extracted and turned into aTags serialized in Turtle format. For this trial, each aTag was annotated with the MeSH terms associated with the article. In future work, this could be enhanced or replaced with annotations created by automated entity recognition (e.g., BioPortal webservice or EBI Whatizit) or manual curation.

## Competing interests

No competing interests declared.

## Authors' contributions

MS is leading the aTag project, developed the aTag applications and created the aTag datasets. HS assisted with programming and web design.
